# Proinflammatory Cytokine Expression in Apical Periodontitis from Diabetic Patients

**DOI:** 10.1155/2023/4961827

**Published:** 2023-02-10

**Authors:** Estéfano Borgo Sarmento, Rosana Helena Teixeira de Lima Ribeiro Andrade, Cinthya Cristina Gomes, Fábio Ramôa Pires, Maria Isabel Bastos Valente, Adalgiza Mafra Moreno, Marco Orsini, Dennis Carvalho Ferreira, Lucio Souza Gonçalves, Luciana Armada

**Affiliations:** ^1^Postgraduate Program, School of Dentistry, Fluminense Federal University, Nova Friburgo, Rio de Janeiro, Brazil; ^2^Postgraduate Program in Dentistry, Estácio de Sá University, Rio de Janeiro, Brazil; ^3^Iguaçu University, Nova Iguaçu, Rio de Janeiro, Brazil

## Abstract

Diabetes mellitus has been regarded as a condition capable of influencing the evolution of periapical lesions. Therefore, this study evaluated the immunoexpression of IL-1*β*, TNF-*α*, and IL-17 in apical periodontitis from type 2 diabetic patients through immunohistochemistry. Twenty-six periapical lesions were selected, and the images obtained through immunohistochemistry reactions were analyzed. The statistical significance was set at *p* < 0.05. IL-1*β* expression was considered focal (54%), weak to moderate (38%), and strong (8%) in diabetics, and focal (46%), weak to moderate (31%), and strong (23%), in controls. TNF-*α* was focal (85%) and weak to moderate (15%) in diabetics, and focal (92%) and weak to moderate (8%), in controls. IL-17 was focal (8%), weak to moderate (46%), and strong (46%) in diabetics, and focal (62%), weak to moderate (8%), and strong (30%), in controls. The quantitative analysis revealed greater expression of IL-17, with a significant difference between IL-17 × IL-1*β* × TNF-*α*(*p*=0.0009) in the diabetic group. The cytokines IL-1*β* and TNF-*α* did not express statistical differences between the tested groups. The IL-17 showed higher immunoexpression in the diabetic group (*p*=0.047), which may suggest higher bone resorption activity in chronic apical periodontitis in this group of patients.

## 1. Introduction

Apical periodontitis (AP), characterized by resorption of alveolar bone around the root apex, is an inflammatory and immune reaction of periapical tissues in response to root canal infection [1, 2]. The occurrence of periradicular pathologies is associated with the immune host factors that can act directly on the severity of the disease [3].

In order to neutralize the pathogens, the immune response is characterized by the recruitment of inflammatory cells and the production of cytokines and chemokines [4]. Macrophages, T lymphocytes, and other types of cells release chemical mediators, such as *interleukin 1β* (IL-1*β*), *tumor necrosis factor-alpha* (TNF-*α*), *interleukin 17* (IL-17), prostaglandins, and bradykinins, which on reaching high levels indirectly activates the osteoclastogenesis process [5] and can play a fundamental role in the bone resorption associated with apical periodontitis.

IL-1*β* is a proinflammatory cytokine that belongs to the IL-1 family, secreted by several cells, including monocytes, macrophages, and fibroblasts [6]. It has a fundamental role in many chronic diseases and is known to be one of the main stimulators of bone resorption [4].

Cells belonging to the T helper (Th) lineage, such as Th1, increase the inflammatory process by secreting cytokines such as TNF-*α* that activate osteoclastogenesis and polymorphonuclear cells [7]. The cytokines released by Th17, such as IL-17 and IL-23, are considered proinflammatory and may be associated with increased bone resorption in AP [8].

On the other hand, some systemic conditions of individuals can modify the course of the disease and allow a different response to infection. Such conditions can influence the development, diagnosis, severity, or even response to treatment [1, 9]. Disorders such as diabetes mellitus are capable of being associated with increased susceptibility to infections [10, 11], a deficiency of the immune response [12], increased bone resorption [13], and consequently, a higher prevalence of radiolucent periapical lesions [14, 15].

Therefore, based on the hypothesis that type 2 diabetic individuals may present a greater expression of proinflammatory cytokines involved in bone resorption, this study aimed to compare the immunoexpression of IL-1*β*, TNF-*α*, and IL-17, in periapical lesions from type 2 diabetic and normoglycemic patients. We hope to clarify the influence of type 2 diabetes on the expression of these chemical mediators in periapical lesions since this type of study is still scarce in the literature.

## 2. Materials and Methods

This case-control study was approved by Ethical Committee for Research and followed the strengthening the reporting of observational studies in epidemiology (STROBE statement).

The samples evaluated were collected from January 2018 to December 2019 and consisted of 26 periapical lesions (13 study group and 13 controls) from teeth with clinical and radiographic diagnosis of pulp necrosis and primary chronic apical periodontitis indicated for extraction.

Samples were excluded from this study when they were: from patients with other systemic factors or acquired habits (such as hypertension and tabagism), from patients who made use of medicaments (such as analgesic, anti-inflammatory, and/or antibiotic) 3 months before surgery, from teeth with endodontic-periodontal infections, previous endodontic treatment, and root fractures.

Furthermore, the blood glucose levels of all patients were assessed by laboratory tests. All individuals in the study group stated that their glycemia was controlled.

Anamnesis, clinical, and laboratory examinations were used to acquire demographic data, the location and position of the tooth in the dental arch (anterior or posterior), medications in use, and glycemia and glycated hemoglobin values (HbA1c).

The following values were considered to assess HbA1c levels: up to 5.7% of total Hb in the normoglycemic, 5.7–6.4% in individuals with a higher risk for diabetes mellitus, and above 6.4% in diabetics [16]. Therefore, our sample was composed only of individuals with a glycated hemoglobin index above 6.4% in the diabetic group and below 5.7% in the normoglycemic group. All individuals were submitted blood testing (fasting glycemia and HbA1c) before the surgical procedure [17].

### 2.1. Histopathological Diagnosis of Periapical Lesions

Lesions were removed after dental extraction and stored in 10% buffered formalin. After that, tissues were prepared to inclusion in paraffin and histological sections [18].

The histopathological analysis of hematoxylin-eosin stained histological slides was performed to diagnosis of the periapical lesion by two evaluators.

### 2.2. Immunohistochemical Reactions

Histological sections were mounted on silanized slides to perform the reactions according to a previously described protocol [8, 19]. The following primary antibodies were used: IL-1*β* (1 : 100, rabbit, sc-7884), IL-17 (1 : 200, rabbit, sc-7927), and TNF-*α* (1 : 50, mouse, sc-130349) from Santa Cruz Biotechnology (Dallas, TX). The secondary antibody LSAB + HRP system (Dako K0690, DAKO North America, and Carpinteria, CA) was used. To stain the areas of cytokine expression, Liquid DAB (Dako K3468, DAKO North America, and Carpinteria, CA) was used. Following the manufacturers' instructions, positive and negative controls were used for each antibody.

### 2.3. Image Analysis

Two calibrated evaluators analyzed the images separately with an optical microscope (Leica DM500, Heerbrugg, Sweden), and each slide was subdivided into 5 high magnification fields (40x microscopic magnification) [8, 19].

Values from 0 to 2 were assigned to each field, considering the number of positive markings. The areas observed were negative to focal: no positive cells or <5% of the cells stained positively (0 points); weak to moderate, 5% to 50% of the cells (1 point); and strong, >50% of the cells (2 points).

Lastly, each zone of all the slides studied received five degrees, which together represented values from 0 (negative) to 10 (strongly positive). As this final number summarizes the total value of the five fields, an average was obtained referring to the immunoexpression classification, which was: negative to focal (0 to 0.5), weak to moderate (0.6 to 1.2), and strong (1.3 to 2.0).

### 2.4. Statistical Analysis

The GraphPad Prism 6 (GraphPad Software, Inc., San Diego, CA, USA) was used to analyze the data. The normality of the quantitative variables was tested by using the Kolmogorov–Smirnov and Shapiro–Wilk tests and by graphical analysis. The nonparametric Mann–Whitney test was used for age, HbA1c values, and comparison of the expression of each mediator between the groups, whereas the Kruskal–Wallis test and Dunn's multiple comparison were performed for the comparisons among the three proinflammatory cytokines. The Fisher's exact test evaluated the parameters of the qualitative variables (gender, type of lesion, location, and position in the arch) between the two groups. The statistical significance was set at *p* < 0.05.

## 3. Results

The demographic evaluation of the cases included in the study showed that the mean age between the study group (53.69 ± 9.54) and controls (55.00 ± 12.48) no statistically significant difference (*p* > 0.05), where the youngest was 36 years old and the oldest was 76 years old. Regarding gender, in the diabetic group 38% males and 62% females and in the control group 15% males and 85% females (*p* > 0.05).

The diabetic group showed mean HbA1c (6.9% ± 0.70) significantly higher than the control group (5.1% ± 0.26) (*p*=0.0001).

The histological analysis of lesions indicated that 69% of the samples were classified as granulomas and 31% as cysts in both groups. The radiographical analysis of the lesions showed that 85% in the maxilla and 15% in the mandible in the diabetic group, and 69% in the maxilla and 31% in the mandible in the control group. In the diabetic group, 46% were in the anterior position and 54% in the posterior position, and in the control group, 38% in the anterior position while 62% were in the posterior position. There was no significant difference between these variables (*p* > 0.05) (Table 1).

**Figure 1:**
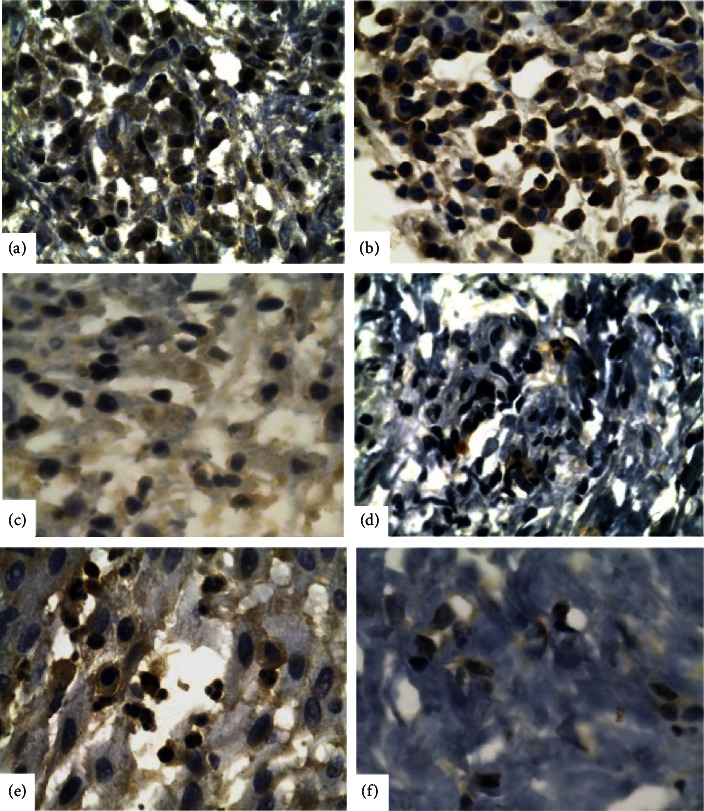
Photomicrographs of histological sections with the positive marking for proinflammatory cytokines in apical periodontitis lesions (immunoperoxidase; 40x). Diabetic patients-(a) IL-1*β*, (b) IL-17, and (c) TNF-*α*. Control patients-(d) IL-1*β*, (e) IL-17, and (f) TNF-*α*.

The qualitative analysis of proinflammatory cytokines in the images obtained through immunohistochemistry in the diabetic group (Figures 1(a)–1(c)) revealed that the expression of IL-1*β* were 54% focal, 38% weak to moderate, and 8% strong; TNF-*α*: 85% focal and 15% weak to moderate; IL-17 : 8% focal, 46% weak to moderate, and 46% strong. There were no negative samples for the evaluated markers. The analysis of the control group (Figures 1(d)–1(f)) revealed that the expression of IL-1*β* was 46% focal, 31% weak to moderate, and 23% strong; TNF-*α*: 92% focal and 8% weak to moderate; IL-17 : 62% focal, 8% weak to moderate, and 30% strong. There were also no negative samples for the evaluated markers.

The quantitative analysis revealed a greater expression of IL-17, and there was a significant difference between IL-17 × IL-1*β* × TNF-*α*(*p*=0.0009) in the diabetic group. There was no significant difference in the control group (*p*=0.677) (Table 2).

**Figure 2:**
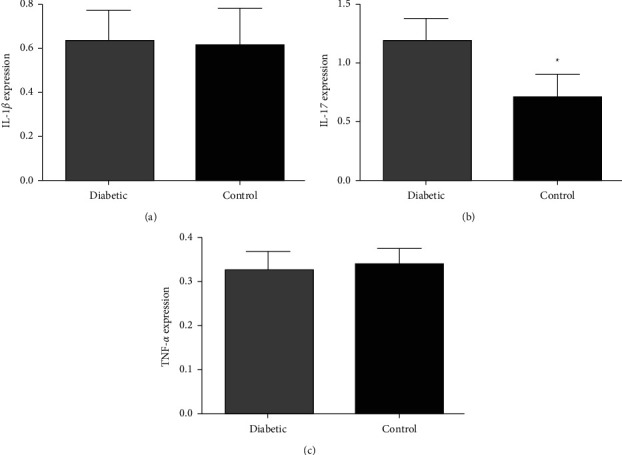
Comparison between the proinflammatory cytokines expressions in periradicular lesions of diabetic patients with control patients (Mann–Whitney test). (a) IL-1*β*, (b) IL-17, and (c) TNF-*α*. ^*∗*^*p* < 0.05.

The expression of cytokines in the apical periodontitis lesions in the diabetic and control groups (Figure 2) had similar results, except for the greater expression of IL-17 (*p*=0.047) in the diabetic group.

## 4. Discussion

Although the role of cytokines (proinflammatory and anti-inflammatory) in the development and repair of periapical lesions has already been demonstrated [20], little is known about the influence of disease modifiers, such as type 2 diabetes mellitus, on the immunoexpression of these chemical mediators in periapical lesions.

This study compared the immunoexpression of IL-1*β*, TNF-*α*, and IL-17 in periapical lesions obtained from diabetic and normoglycemic individuals. The results revealed an increase of IL-17 expression, which may cause an increase in the bone resorption process. However, IL-1*β* and TNF-*α* show no statistical difference between the diabetic and normoglycemic groups.

The methodologies used for the immunohistochemical reactions and the assessment of the cytokine expressions was based on previous studies [8, 19, 21].

When analyzed statistically, the results obtained regarding location of the periapical lesion, age, and gender did not reveal any significant differences between the groups, demonstrating that the samples are comparable, which is in agreement with the results of previous studies [11, 22].

The HbA1c index was evaluated in all participants. The diabetic group had significantly higher values than the control group, which confirmed that all individuals in the experimental group had diabetes mellitus. The validation of the individual's systemic status before the surgical procedure provides a better basis for the results.

Histological and radiographic evaluations revealed high quantities of granulomas in both groups, most of them located in the maxilla in the posterior position. These variables did not reveal significant differences, as well as shown by a previous study performed on patients without systemic impairment [8].

The IL-1*β* expressed high percentages of focal and weak to moderate markings in both groups, characterizing the chronic inflammatory process present in periapical lesions. This result corroborates with a previous study [23] in which the expression of IL-1*β* was observed close to many osteoclasts during the acute phase of the lesion; however, during the chronic phase, this expression was significantly reduced.

The results of the expression of TNF-*α* in this study are confirmed by other studies that reinforce the participation of this cytokine in periradicular inflammatory processes [20, 24]. It is believed that the presence of TNF-*α* causes the progression of lesions and induces bone resorption through the activation of local osteoclasts [24]. The lower immunoexpression of this proinflammatory cytokine found in our study may be compatible with the chronic phase of AP [25].

The analysis of cytokines revealed higher expression of IL-17 in the diabetic group, showing a statistically significant difference between the groups. This difference may have occurred because IL-17 is involved in both the progression of localized chronic infections (such as chronic apical periodontitis) and in severe systemic diseases (such as diabetes mellitus) [8, 26].

The increase in the immunoexpression of this cytokine in AP can be explained by a large amount of bacteria present in the periapical region, causing a greater release of inflammatory mediators [27, 28]. In the mechanism of development of these lesions, the IL-17 activates many of the cytokine signaling events and is considered an important link molecule between the innate and adaptive immune system [26]. It also has a significant role in the regulation of neutrophil function [29].

Evidence indicates that the presence and activation of the IL-17 in apical periodontitis can stimulate the production of other inflammatory cytokines that unbalance osteoclastogenesis and enables periapical bone destruction [26].

According to Cintra [30], in diabetic individuals the increase of IL-17 may be associated with oxidative stress and advanced glycation end products (AGES) that can promote conditions that exacerbate inflammation. The hyperglycemic state caused by diabetes provides an accumulation of AGES [12], this accumulation in tissues and interaction with the receptor for advanced glycation end products (RAGE), expressed in greater amounts in diabetic cases, can lead to the higher expression of proinflammatory cytokines. The length of time an individual has had diabetes and a high concentration of glucose in the body too contributes to the formation of AGES [12]. In the present study, all patients reported more than 5 years of the disease. For this reason, diabetics likely showed an exacerbated inflammatory response characterized by an increased expression of IL-17.

A limitation observed in the present study was the small sample size. But, this sample size represents the individuals included in the proposed period who met the strict eligibility criteria for conducting the study. Despite the small sample size, the qualitative evaluation of the expression of biomarkers in the immunohistochemical reactions confirmed the presence of these cytokines in the development of periapical lesions since there were no negative samples for these markers.

## 5. Conclusions

In conclusion, the findings of the present study suggest that individuals with diabetes mellitus may present an increase of IL-17 in the area of the periapical lesion, which may cause an increase in the bone resorption process. However, IL-1*β* and TNF-*α* show no statistical difference between the diabetic and normoglycemic groups.

Therefore, further studies involving immunocompromised individuals and the expression of biomarkers involved in the processes of inflammation and bone resorption should be developed in order to understand the likely links between the evolution of apical periodontitis and the systemic health of diabetic patients.

## Figures and Tables

**Table 1:** Distribution of different variables from the diabetic and control groups.

	Diabetics (%)	Controls (%)	*p* values
Type of lesion	9 granulomas (69) 4 cysts (31)	9 granulomas (69) 4 cysts (31)	1.00

Location	11 maxilla (85) 2 mandible (15)	9 maxilla (69) 4 mandible (31)	1.00

Position in the arch	6 anterior (46) 7 posterior (54)	5 anterior (38) 8 posterior (62)	0.64

The parameters were evaluated using the Fisher's exact test.

**Table 2:** Comparison between proinflammatory cytokines in apical periodontitis lesions.

Cytokine	Expression	*p* values
Diabetics		
IL-1*β*	0.63 ± 0.50	0.0009
IL-17	1.20 ± 0.65	
TNF-*α*	0.32 ± 0.15	

Controls		
IL-1*β*	0.61 ± 0.40	
IL-17	0.70 ± 0.72	0.667
TNF-*α*	0.33 ± 0.12	

The parameters were evaluated using the Kruskal–Wallis and Dunn's multiple comparison nonparametric tests. Values are expressed as mean ± standard deviation.

## Data Availability

The data used to support the findings of this study are included within the article.
